# Drug liking and wanting, not impulsive action or reflection is increased by 4-fluoroamphetamine

**DOI:** 10.1007/s00213-018-4931-7

**Published:** 2018-05-31

**Authors:** K. P. C. Kuypers, E. B. de Sousa Fernandes Perna, P. C. Dolder, S. W. Toennes, E. L. Theunissen, N. L. Mason, N. R. P. W. Hutten, J. G. Ramaekers

**Affiliations:** 1 Department of Neuropsychology and Psychopharmacology, Faculty of Psychology and Neuroscience, Maastricht University, P. O. Box 616, 6200 MD Maastricht, the Netherlands; 2 Department of Forensic Toxicology, Institute of Legal Medicine, Goethe University of Frankfurt, Frankfurt, Germany

**Keywords:** 4-FA, Impulsive reflection, Impulsive action, Drug liking, Drug wanting, Good drug effect

## Abstract

**Background:**

New psychoactive substances (NPS) are chemical analogues designed to mimic the effects of various classic recreational drugs of abuse including MDMA, LSD, and cannabis. NPS use is associated with concern about the acute and longer-term effects particular substances might have, with abuse and addiction as potential consequences. Impulsivity and sensitivity to the rewarding effects of drugs have been considered as risk factors for drug abuse. In light of the popularity of 4-fluoroamphetamine (4-FA), it is important to assess whether 4-FA can lead to subjective drug liking and wanting, and impulsive behavior, all factors contributing to the abuse likelihood of a substance.

**Methods:**

A placebo-controlled 2-way crossover study in 12 healthy poly-drug using participants was conducted to test subjective and behavioral effects of 4-FA (100 mg). 4-FA concentrations were determined in serum up to 12 h after administration and two impulsivity tasks and two drug experience questionnaires assessing drug liking and wanting, and good and bad drug effect, were administered between 1 and 11 h post-administration.

**Results:**

Findings showed that 4-FA did not affect impulsive behavior. Self-ratings of drug liking and wanting and good drug effect were increased 1 h after administration; this effect was absent 11 h after drug intake.

**Discussion and conclusion:**

To conclude, 4-FA (single dose) increased self-rated *liking and wanting*, which is known to contribute to the abuse likelihood of a substance; however, it left another factor *impulsive behavior* unaffected. It has to be noted that the current picture is limited and might change with increased sample size, and/or different 4-FA doses.

**Clinical trial registration:**

Trial acronym: 4-FA. URL: http://www.trialregister.nl/trialreg/admin/rctview.asp?TC=6164. Registration number: NTR6164 (Dutch clinical trial registry number).

## Introduction

New psychoactive substances (NPS) are chemical analogues designed to mimic the effects of various classic recreational drugs of abuse including MDMA, LSD, and cannabis (EMCDDA 2015). Anecdotal evidence by users suggests that NPS have subjective effects comparable to the classical psychoactive substances though these effects have not been substantiated yet in human experimental studies (Hondebrink et al. 2017; Liechti 2015). Scientific knowledge about NPS’s acute effects is needed, seen their exponential increase in availability and number over the last 10 years. Moreover, this surge has gone hand in hand with an increase in use, and emergency department visits due to over-intoxication (EMCDDA 2015; Wood et al. 2013).

4-Fluoroamphetamine (4-FA) is a prototypical example of an NPS, belonging to the chemical class of *phenethylamines* (Hondebrink et al. 2017). It appeared on the Dutch “drug market” between 2007 and 2009 where after reports of acute toxic effects steadily increased to even 16% of all reported cases on large scale events (Wijers et al. 2017). Although it was first used as an adulterant in drugs such as amphetamine and MDMA, it became a drug of choice, liked by users for its effects (Linsen et al. 2015). While it has become a popular drug with a recent survey amongst Dutch partygoers revealing that a quarter of the respondents between 15 and 35 years of age had used 4-FA in the past year, the majority (80%) also states that their 4-FA use remained limited to just a few times (Monshouwer et al. 2016).

The effects of 4-FA reportedly range between those of amphetamine, a stimulant, and MDMA, an empathogen (Linsen et al. 2015) which is in line with the biological profile that is also suggested to be in between that of MDMA and amphetamine. Studies determining the monoamine transporter and receptor binding profile in animals and human tissue showed that 4-FA has relatively more serotonergic transporter action compared to amphetamine. Next to the promotion of norepinephrine (NE) and dopamine (DA) release, it was shown to release serotonin (5-HT) similarly to MDMA (Nagai et al. 2007; Rickli et al. 2015).

Linked to NPS use is the concern about the acute and potential long(er) term effects the particular substance might have on behavior and cognitive processes (Schifano et al. 2015) with abuse and addiction as potential consequences. It is known that substances with more pronounced action at the DA-transporter (DAT) can have a higher abuse potential compared to substances that increase activity of the 5-HT system (Wee et al. 2005). Furthermore, a high DAT-to-serotonin transporter (SERT) ratio is a pharmacological characteristic predicting more stimulant effects and a higher potential for addiction. The DAT/SERT ratio of 4-FA is approximately five times higher than MDMA and seven times lower than d-amphetamine (Hondebrink et al. 2017; Rickli et al. 2015). Drug abuse and addiction are related to impulse control, with substances disturbing impulse control eventually evolving into drug abuse when the behavior becomes driven by drug-cues (de Wit 2009; Winstanley et al. 2010). Similarly, sensitivity to the rewarding effects of drugs have also been considered as risk factors for drug abuse (Waefer and de Wit 2013).

The most sensitive and reliable measures of abuse likelihood of a substance are self-ratings of drug liking, or the report of how much the user likes the drug (Carter and Griffiths 2009). In addition, the measures wanting, good drug effect, and bad drug effect seem to co-vary with liking (Carter and Griffiths 2009). On the other hand, typical paradigms to assess different components of impulsivity are the matching familiar figures task (MFFT) and the stop-signal task (SST). In the latter SST-paradigm, which tests motor impulsivity or impulsive action, the participant has to respond continuously to stimuli and withhold their pre-potent response to “stop” stimuli (Logan et al. 1984). In the former MFFT-paradigm, which tests cognitive impulsivity or reflection impulsivity, the participant has to match a target figure to one of the six shown “look-a-likes” of which five differ from the original one by only a small detail. The skill is to withhold the reaction until the match is found (D’Amour-Horvat and Leyton 2014; Perales et al. 2009).

In general, it has been shown that amphetamine (10–40 mg) does not affect impulsive action (Dolder et al. 2018), though when it does, an enhancing effect was found only in people who either performed badly at baseline (de Wit et al. 2000; de Wit et al. 2002) or when presented with complex stimuli (Fillmore et al. 2003). Likewise, studies with MDMA have shown that in doses ranging from 25 to 125 mg, MDMA either leads to improvement of behavioral inhibition or induces no change (Bosker et al. 2010; Kuypers et al. 2007; Schmidt et al. 2017). In one single study, MDMA led to an increase in impulsive action and impulsive reflection (van Wel et al. 2012). The effects of amphetamine and MDMA on subjective measures of drug liking also seem to be similar, with an increase in self-rating up to 3 h after administration of a single dose (Cami et al. 2000; Harris et al. 2002; Rush et al. 2001).

In light of the popularity of 4-FA (Monshouwer et al. 2016), it is important to assess whether 4-FA can lead to subjective drug liking and impulsive behavior, two risk factors linked to abuse liability of a substance. Given the similarity to amphetamine and MDMA, it was hypothesized that 4-FA would not lead to effects on impulsive action or reflection and would lead to a subjective state of drug liking and wanting, and good drug effect around peak drug concentrations and not after 4-FA plasma concentrations have decreased substantially.

The data presented in this paper are part of a larger project PREDICT (www.predictnps.eu) focusing at the safety profile of NPS in humans, in vitro and in silico. Additional data of the present study including the safety profile and neurocognitive effects of 4-FA are published in a separate paper (de Sousa Fernandes Perna et al. 2018).

## Methods

### Study design and treatment

The study was conducted according to a two-way crossover, randomized, counter-balanced, and placebo-controlled design. Treatment was 100 mg of 4-FA or placebo mixed with 100 mL of bitter lemon. The drink was ingested at once. The 100 mg dose was based on a user survey amongst Dutch 4-FA users. The majority (75%) of the users who knew which dose they ingest typically indicated it to be between 50 and 150 mg, the remainder used larger doses. In general, the subjective effects last between 4 and 6 h (Linsen et al. 2015). 4-FA has an estimated half-life of 3.7 h in the rat brain (Fuller et al. 1975).

A permit for obtaining, storing, and administering 4-FA was obtained from the Dutch drug enforcement administration. The study was performed in accordance with the Helsinki Declaration of 1975, and its subsequent amendments, and was approved by the Medical Ethics Committee of the Academic Hospital of Maastricht and the University of Maastricht. It was registered in the Dutch Clinical Trial Register (Registration Number: NTR6164).

### Participants

Participants were 12 healthy recreational polydrug users aged 22.3 (± 3.4) years on average (± SD) of whom 7 were male with a mean BMI of 22.9 (± 1.3 SD) and 5 were female with an average BMI of 21.5 (± 2.8 SD). All of them had experience with alcohol use with the units consumed per week ranging from 2 to 20. One participant smoked cigarettes with an average of 15 per day and seven smoked cannabis with an average of 1 “unit” per week. The use of other drugs was expressed in “lifetime use”; the minimum and maximum (min-max) times used, together with the number of participants with experience (N) is listed per drug (N; min-max): amphetamines (8; 1–32), cocaine (6; 1–26), ecstasy (11; 1–35), 4-FA (5; 1–25), LSD (3; 7–17), and other drugs like mushrooms and ketamine (8; 1–33).

### Procedures

Participants were recruited by means of flyers in the university building, an advert on a research-Facebook page, and by word of mouth. When interested, they were sent the information brochure explaining the background, aims and study procedure, and two questionnaires (medical and drug use history). When they were fully informed, potential questions were answered, and if they fulfilled at first sight of the inclusion criteria, they were invited for a medical screening. When no objections were raised during the physical examination including a standard blood and urine screens and an electrocardiogram (EKG) and participants signed the informed consent, they were included in the study.

Inclusion criteria were previous experience with psychostimulants (≤ 1 time/week) and at least one time during the previous year; age between 18 and 40 years; free from psychotropic medication; healthy based on the assessment of medical history, physical examination, vital signs, EKG (with heart rate 51–100 bpm; lower limit for fit people, 45 bpm), a resting systolic blood pressure 91–140 mmHg, a resting diastolic blood pressure 51–90 mmHg, and the results of the hematology, clinical chemistry, urinalysis, and serology within the reference ranges; normal binocular visual acuity, corrected or uncorrected; absence of any major medical, endocrine and neurological conditions, and normal weight as defined by a body mass index between 19.5 and 28 kg/m^2^; written informed consent. Exclusion criteria were history of drug abuse or addiction (determined by the medical questionnaire, drug questionnaire, and medical examination); excessive drinking (> 20 alcoholic consumptions a week); pregnancy or lactation; hypertension (diastolic > 90; systolic > 140); current or history of psychiatric disorder (determined by the medical questionnaire and medical examination); liver dysfunction; (serious) side effects to previous psychostimulant use; history of cardiac dysfunctions (arrhythmia, ischemic heart disease,…); simultaneous participation in another clinical trial; being a blood donor; and for women: not using reliable contraceptive.

After study inclusion and prior to the test days, participants were familiarized with the study procedures, tests, and questionnaires. On a test day, participants arrived early in the morning and they were tested for the absence of drugs in urine and alcohol in breath. In case of females, an additional test for pregnancy was conducted in urine. When all tests were negative, participants were given baseline questionnaires, a blood sample was taken and they received a light-standardized breakfast. Table 1 provides an overview of timing of the questionnaire, the impulsivity tests and blood samples during the test day, which took 12.5 h in total. The test schedule was identical for each test day and each participant. Participants were paid upon completion of the testing periods for their participation.

**Table 1:** Time schedule of the impulsivity paradigms and drug experience questionnaires relative to treatment administration; blood samples were collected more frequently than depicted here, only samples collected around times of tests reported here are shown

	T0	T1	T2	T3	T4	T5 − 1	T5	T5 + 1
Post-treatment	0 h	1 h	2 h30 min	4 h	8 h	10 h	11 h	12 h
Motor impulsivity (SST)		X		X	X			
Reflective impulsivity (MFFT)			X					
Drug liking and wanting (SDRQ)		X					X	
Good and Bad drug effect (POMS)	X	X		X			X	
4-FA serum concentrations		X		X	X	X		X

### Drug experience questionnaires

#### Sensitivity to drug reinforcement questionnaire

The sensitivity to drug reinforcement questionnaire (SDRQ) asks participants to rate their liking and wanting of 4-FA use during their present condition on a 5-point rating scale (1 = somewhat; 2 = slightly; 3 = moderately; 4 = very; 5 = extremely). The questionnaire is comprised of two questions, “How pleasant is using 4-FA right now?” and “How much do you want to use 4-FA right now?” referring respectively to drug liking and drug wanting.

#### Profile of mood states

The Profile of Mood States (POMS) (de Wit et al. 2002) is a self-assessment mood questionnaire with 72 five-point-Likert scale items on which participants have to indicate to what extent these items were representing their mood. Items are clustered to represent eight basic mood states: anxiety, depression, anger, vigor, fatigue, confusion, friendliness, and elation. From those scales, two composite scales were derived, good drug effect (vigor + friendliness + elation/22) and bad drug effect (anxiety + depression + anger + fatigue + confusion/50).

### Impulsivity tests

#### Stop signal test

The current stop-signal test (SST) is adapted from an earlier version of Fillmore and colleagues (Fillmore et al. 2002), it assesses impulsive action and it has previously been used in similar research (Kuypers et al. 2007; van Wel et al. 2012). It requires participants to make quick key responses to visually presented go signals and to inhibit any response when a visual stop signal (an asterisk) is suddenly presented in one of the corners of the screen. This can occur after one of four fixed delays (50, 150, 250, and 350 ms) after the onset of the go signal. The go signals were four letters presented one at a time for 500 ms in the center of a computer screen. Participants are required to respond to each letter as quickly as possible by pressing on of two response buttons. The computer screen is blank for 1.5 s before the next letter is displayed. This provides a period of 2 s in which the participant can respond to the go signal. A single test consists of 176 trials in which each of the 4-letter stimuli will be presented equally often. A stop signal occurs in 48 trials during a test. Participants are required to withhold any response in case a stop signal is presented. The task lasts about 10 min. Dependent variables are proportion of correct go responses and failed inhibitions on stop trials and corresponding reaction times (Logan et al. 1984). The Stop reaction time (stop RT) to stop signal represents the estimated mean time required to inhibit a response.

The method for calculating stop reaction time was taken from the race model of inhibitory control (Logan 1994). This model proposes that the response to stop signals is defined by two parallel processes: execution of a motor action in response to a signal and inhibition of a motor action in response to a stop signal. Crucial to the outcome of the race is the speed of both processes. Response inhibition will fail if the time required to inhibit exceeds the time to complete a motor response at the time of the stop signal.

The speed of the inhibition response cannot be observed directly but can be derived mathematically on the basis of three factors: stop-signal delay, reaction time distribution on go trials, and the probability of successful response inhibitions in stop signal trials. First, reaction times to 128 go trials were rank ordered from shortest to longest. The finishing time of the inhibition response was then determined from the probability of successful response inhibition and the distribution of reaction times. If *n* percent of the responses on stop-signal trials would be unsuccessfully inhibited (failed inhibitions), then the finishing time would be associated with the *n*th percentile of the RT distribution. Stop RT was then determined by subtracting the appropriate stop-signal delay from reaction time at the *n*th percentile of the RT distribution. The resulting values for each stop signal delay were then averaged to yield a single measure of stop reaction time for the test.

### Matching familiar figures test

The matching familiar figures test (MFFT) assesses impulsive reflection, which is the tendency to reflect on the validity of problem solving under the special condition of several possible alternatives. The test involves simultaneous presentation of a target figure positioned on the left of the screen and an array of six alternatives on the right half of the screen, all except one differing in one or more details from the target figure. The participant is asked to select from the alternatives the figure that exactly matches the target as quickly as possible. If the initial selection is incorrect, this is signaled with a beep and subjects are required to give another answer. Each participant is given 2 examples followed by 20 test items. The response latency and number of errors before the correct match are collected per item. The main dependent variables resulting from these measures are the mean latency for first response, the accumulated number of errors made before the correct match, an impulsivity score (I-score), and an efficiency score (E-score). The I-score is a composite index of impulsivity, whereas the E-score reflects the balance between “fast and accurate” and “slow and inaccurate.” The I-score is calculated by subtracting the standardized mean latency from the standardized number of errors. The E-score is calculated by adding the standardized mean latency to the standardized number of errors and multiplying the result by − 1 (Perales et al. 2009).

### Pharmacokinetics

A blood sample (5 mL) was collected at baseline and at regular times after treatment (see Table 1). Samples were centrifuged immediately and resulting serum was pipetted into a clean tube and stored at − 20 °C until 4-FA concentration determination which took place after study completion. Blood serum (0.5 mL) was diluted with buffer and internal standard solution was added. After liquid-liquid extraction the extract was analyzed using LC-MSMS, with 0.04 ng/mL as the lower limit of quantification.

### Statistical analyses

Questionnaire data and data of the SST was analyzed with repeated measures general linear models (RM GLM) ANOVA with treatment (two levels) and time of measurement (two levels SDRQ, three levels POMS and SST) as within subject factors (SPSS, version 24.0). In case of main effects of time of measurement, Bonferroni-corrected post-hoc tests were conducted. Data of the MFFT was analyzed by means of paired samples *t* tests since there was only one assessment. The alpha criterion level of statistical significance for all analyses was set at *p* = 0.05. Partial eta squared (*ƞ*_*p*_^2^) is reported in case of significant effects in the ANOVA GLM to demonstrate the effect’s magnitude, where 0.01 is defined as small, 0.06 as moderate and 0.14 as large. Partial eta squared is based on Cohen’s *f* which defines small, medium and large as respectively 0.10, 0.25, and 0.50 which corresponds to *η*^2^ of 0.0099, 0.0588, and 0.1379 (Richardson 2011).

## Results

### Pharmacokinetics

Mean (± SE) 4-FA serum concentrations were 167.3 ng/mL (±15) at T1, 60′ post-treatment, peaked 2 h after intake (205.4 ng/mL ± 45) and descended over time to 97.2 ng/mL (± 10), 12 h after 4-FA administration (T5 +1).

### Drug experience questionnaires

#### Sensitivity to drug reinforcement questionnaire

**Fig. 1:**
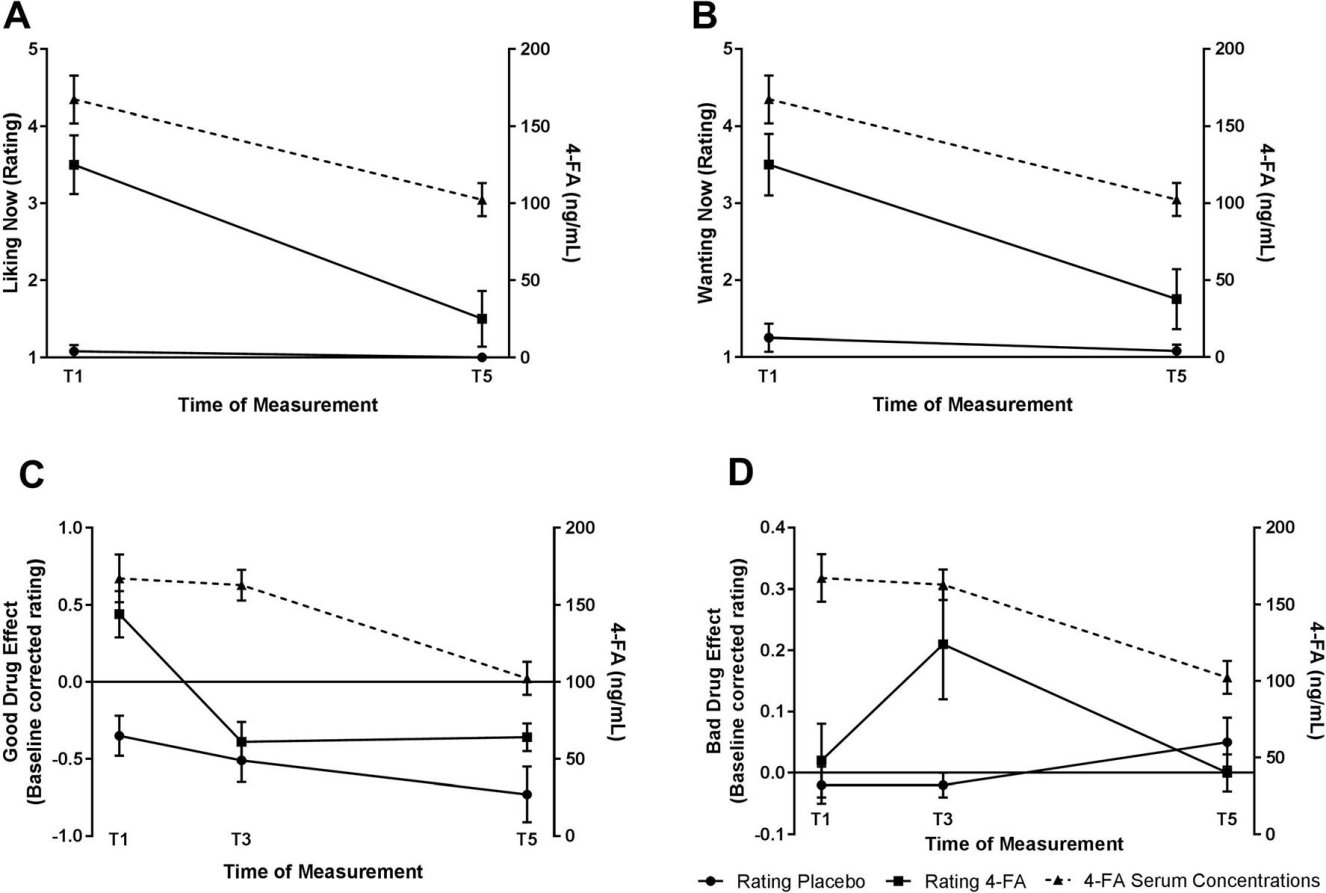
Mean (± SE) ratings of 4-FA liking (**a**) and 4-FA wanting (**b**) 1 and 11 h after treatments, and ratings of good drug effect (**c**) and bad drug effect (**d**) 1, 4, and 11 h after treatments and corresponding 4-FA serum concentrations

RM GLM ANOVA showed statistically significant effects of Treatment, Time and Treatment by Time on both scales of the SDRQ. Ratings of liking were higher after 4-FA compared to placebo (*F*_1,11_ = 26.16; *p* < 0.001; *ƞ*_*p*_^2^ = 0.70), they were highest at T1 compared to T5 (*F*_1,11_ = 22.99; *p* = 0.001; *ƞ*_*p*_^2^ = 0.68), and while the liking ratings remained stable in the placebo condition, they decreased substantially in the 4-FA condition over time (*F*_1,11_ = 13.13; *p* = 0.004; *ƞ*_*p*_^2^ = 0.54) (Fig. 1a). For wanting the same pattern was observed with higher ratings of wanting after 4-FA compared to placebo (*F*_1,11_ = 19.06; *p* = 0.001; *ƞ*_*p*_^2^ = 0.63), highest ratings at T1 compared to T5 (*F*_1,11_ = 14.73; *p* = 0.003; *ƞ*_*p*_^2^ = 0.57), and while ratings of wanting remained stable in the placebo condition, they substantially decreased in the 4-FA condition over time (*F*_1,11_ = 22.18; *p* = 0.001; *ƞ*_*p*_^2^ = 0.67) (Fig. 1b).

#### Profile of mood states

Since one of the POMS sub-scales included in the composite scales displayed a baseline difference between test days, baseline-corrected scores entered the analyses. RM GLM ANOVA showed main effects of treatment (*F*_1,10_ = 7.55; *p* = 0.02; *ƞ*_*p*_^2^ = 0.43) and time (*F*_2,20_ = 17.81; *p* < 0.001; *ƞ*_*p*_^2^ = 0.64) and a treatment by time interaction effect (*F*_2,20_ = 8.12; *p* = 0.003; *ƞ*_*p*_^2^ = 0.45) on good drug effect. The good drug effect was higher after 4-FA compared to placebo; the overall effect was the highest on T1 compared to T3 and T5. The quadratic interaction (*F*_1,10_ = 12.57; *p* = 0.005; *ƞ*_*p*_^2^ = 0.56) between treatment and time demonstrated that while the ratings in the placebo condition were low and decreased slightly over time, the ratings in the 4-FA condition were very pronounced at T1 and steeply decreased from T1 to T3 while remaining at the same low level at T5 compared to T3 (Fig. 1c).

Analyses showed a significant treatment by time interaction effect (*F*_1,10_ = 6.59; *p* = 0.006; *ƞ*_*p*_^2^ = 0.43) on bad drug effect. Post-hoc analyses showed that this was a quadratic effect (*F*_1,10_ = 2.19; *p* = 0.02; *ƞ*_*p*_^2^ = 0.43) with the highest rating showing at T3 for 4-FA compared to the other time-points and placebo; this effect was probably driven by the statistically significant increased levels of fatigue and confusion, two of the sub-scales included in this composite scale bad drug effect which were the highest at this time-point. There was no main effect of treatment (*F*_1,10_ = 2.19; *p* = 0.02; *ƞ*_*p*_^2^ = 0.43) or time on bad drug effect (Fig. 1d).

### Impulsivity tasks

#### Stop signal task

One participant was excluded from the analysis because of an absence of responses on go trials on four occasions (three times placebo condition, once 4-FA). Repeated measures GLM ANOVA demonstrated a main effect of time of measurement (*F*_2,20_ = 3.57; *p* = 0.05; *ƞ*_*p*_^2^ = 0.26) and a treatment by time of measurement interaction effect (*F*_2,20_ = 3.52; *p* = 0.05; *ƞ*_*p*_^2^ = 0.26) on the proportion of failed inhibitions. Post-hoc tests did not reveal statistically significant differences between separate time of measurements or treatment by time of measurement performances. Inspecting the data visually led to the suggestion that these effects were mainly driven by the high number of failed inhibitions 1 h after 4-FA administration while the number of inhibition failures in the placebo condition was lower and stable over time. The number of failed inhibitions 4 and 8 h after 4-FA administration was comparable to placebo-levels. Analyses did not reveal statistically significant main effects of treatment or time of measurement, or their interaction on proportion of correct go responses, go-RT or stop-RT (Table 2).

**Table 2:** Mean (± SE) of dependent variables of the SST *F*-, *p*-, and partial eta^2^- values of RM GLM ANOVA

		Mean (± SE)		RM GLM ANOVA, main and interaction effects								
		Treatment		Treatment			Time			Treatment by time		
Stop signal task	T	PLA	4-FA	*F*_1,10_	*p*	*ƞ*_*p*_^2^	*F*_2,20_	*p*	*ƞ*_*p*_^2^	*F*_2,20_	p	*ƞ*_*p*_^2^
Failed inhibitions (%)	T1	0.35 (0.06)	0.47 (0.10)	0.31	0.59	0.03	3.57	0.05	0.26	3.52	0.05	0.26
	T3	0.35 (0.06)	0.32 (0.06)									
	T4	0.34 (0.06)	0.33 (0.06)									
Stop-RT	T1	286 (15)	327 (26)	1.63	0.23	0.14	2.25	0.13	0.18	1.36	0.28	0.12
	T3	291 (21)	287 (12)									
	T3	266 (10)	291 (12)									
Correct go’s (%)	T1	0.70 (0.01)	0.69 (0.01)	0.44	0.52	0.04	2.72	0.09	0.21	1.15	0.33	0.10
	T3	0.67 (0.01)	0.69 (0.02)									
	T4	0.69 (0.01)	0.70 (0.01)									
Go-RT (ms)	T1	569 (42)	554 (42)	0.13	0.72	0.01	1.47	0.25	0.13	2.78	0.09	0.22
	T3	571 (44)	583 (39)									
	T4	558 (38)	584 (44)									

#### Matching familiar figures test

Paired samples *t* tests did not reveal statistically significant differences between 4-FA and placebo on the dependent variables mean latency of first response (*t*_1,11_ = − 0.64; *p* = 0.53) and errors (*t*_1,11_ = 0.12; *p* = 0.9), or the two composite score, impulsivity (*t*_1,11_ = 0.72; *p* = 0.48) and efficiency (*t*_1,11_ = 0.27; *p* = 0.79). Mean (± SE) scores after placebo and 4-FA were respectively 13 (1) and 15 (2) for latency in seconds, 4.00 (1.1) and 3.75 (1.5) for total number of errors, 0.2 (0.3) and − 0.2 (0.5) for I-score, and 0.1 (0.4) and − 0.1 (0.5) for E-score.

## Discussion

The present study aimed to assess whether 4-FA elicits risk factors for drug abuse, namely impulsive reflection and action, a subjective state of drug liking and wanting, and good versus bad drug effect. It was hypothesized that 4-FA would not lead to effects on impulsive behavior but would produce a state of drug liking at peak drug concentrations. As expected, findings showed an absence of 4-FA effects on impulsive reflection and action and an increase in self-ratings of drug liking, drug wanting and good drug effect, 1 h after administration and a peak in bad drug effect 4 h after intake. The liking, wanting, and good drug effects were absent 11 h after drug intake.

The absence of drug effects on the impulsivity measures was in line with expectations and previous studies with amphetamine, MDMA and cocaine (Bosker et al. 2010; de Wit et al. 2000; de Wit et al. 2002; Dolder et al. 2018; Kuypers et al. 2007; Schmidt et al. 2017). The average scores during drug and placebo conditions were also comparable to those found in previous drug studies by our group using the same paradigms (e.g., (Kuypers et al. 2007; van Wel et al. 2012). Although present findings suggest that a single dose of 4-FA (100 mg) does not induce impulsive behavior, previous studies have shown that personal characteristics, like baseline performance and task-related characteristics like stimulus/response complexity, can play a role in drug-induced changes. People performing badly at baseline or who were presented with complex stimuli showed an enhancing effect after amphetamine (de Wit et al. 2000; de Wit et al. 2002; Fillmore et al. 2003). Additionally, stimulants like amphetamine and cocaine have shown to exert enhancing effects on response control in individuals with impulse control problems, like ADHD and drug addiction (Arnsten 2006; Fillmore et al. 2002, 2005).

Self-ratings of liking, wanting, and good drug effect, all reliable and sensitive indicators of drug abuse likelihood, were increased compared to placebo 1 h after intake. Ten hours later, the self-ratings in the 4-FA condition were indistinguishable from ratings in the placebo condition. Findings demonstrate that a single dose of 4-FA (100 mg) does not lead to craving (wanting) when the good drug effects are subsiding and bad drug effects increasing, suggesting an absence of repeated or compulsive use of this drug at this dose. However, similar to behavioral performance, studies have shown that personal or biological factors like baseline performance or DA receptor availability can play a role in subjective drug experience (Brewer and Potenza 2008; McCloskey et al. 2010). It was demonstrated previously that participants who performed worse on an attention paradigm-liked amphetamine (20 mg) less and reported smaller increases in wanting compared to participants who exhibited better attentional capacities. It was concluded that participants’ attention capacities determined the sensitivity to stimulant-induced effects with worse capacity signaling reduced sensitivity to stimulant-induced euphoria (McCloskey et al. 2010). In addition, low baseline measures of D2 receptor availability in non-addicted people was shown to predict methylphenidate liking and high levels of impulsivity in rats (Brewer and Potenza 2008).

While the findings of the present study suggest that a single dose of 4-FA (100 mg) does not lead to either impulsive action or reflection, or to liking and wanting of the drug when the peak effects have subsided, it has to be noted that the current picture is limited and might change with increased sample size, including participants with poor baseline impulse control and attention capacity, and/or different 4-FA doses. Furthermore, additional repetitions of self-rated liking and wanting are needed to know whether these feelings are present when for example bad drug effects are high and good drug effects are low, a few hours after drug intake, as this could push the individual to repeated drug use.
